# Frequency of Acute Stress Disorder (ASD) and Postnatal Depression in Mothers Of Preterm Neonates Hospitalized in a Neonatal Intensive Care Unit (NICU) in Pakistan

**DOI:** 10.12669/pjms.42.2.13932

**Published:** 2026-02

**Authors:** Saima Fayyaz, AS Hussain, Maheen Choudhry, Vardah Noor Ahmed Bharuchi

**Affiliations:** 1 Saima Fayyaz, FCPS Department of Paediatrics & Child Health, The Aga Khan University Hospital, Karachi, Pakistan; 2 AS Hussain, FCPS Department of Paediatrics & Child Health, The Aga Khan University Hospital, Karachi, Pakistan; 3 Maheen Choudhry Department of Paediatrics & Child Health, The Aga Khan University Hospital, Karachi, Pakistan; 4 Vardah Noor Ahmed Bharuchi Department of Paediatrics & Child Health, The Aga Khan University Hospital, Karachi, Pakistan

**Keywords:** Acute Stress Disorder, Postnatal depression, Preterm neonates, Mothers, Neonatal Intensive Care Unit

## Abstract

**Objective:**

To determine the frequency of Acute Stress Disorder (ASD) and postnatal depression in mothers of preterm neonates hospitalized in the neonatal intensive care unit (NICU) of the Aga Khan University Hospital.

**Methodology:**

It is a cross-sectional study. It was conducted in a 24-bed level-3 NICU at a tertiary care hospital in Karachi. Mothers of preterm newborns under 34 weeks’ gestational age admitted to the NICU were included in the study.

**Results:**

Among 123 postpartum women, 43.1% (n=53) met criteria for Acute Stress Disorder (ASD), with significantly lower gestational age in this group: median 29.9 weeks (IQR: 27.6-31.0) vs. 33.0 weeks (IQR: 32.0-33.0) in non-ASD cases (difference in medians: 3.1 weeks, 95% CI: 2.1-4.0). Infants of mothers with ASD had a less mean birth weight (1.3 ± 0.4 kg) compared to those without ASD (1.8 ± 0.5 kg), with a mean difference of 0.5 kg (95% CI: 0.34-0.66). Median NICU stay was significantly prolonged in the ASD group: 20.0 days (IQR: 12.0-28.0) versus 10.0 days (IQR: 7.0-15.0), with an estimated median difference of 10.0 days (95% CI: 6.0-14.0). A higher number of mothers with a miscarriage history (n=23/53, 43.4%) experienced ASD compared to those without (n=17/70, 24.3%), yielding a risk difference of 19.1% (95% CI: 4.3%-33.8%). Mothers with ASD also reported increased Edinburgh Postnatal Depression Scale scores (median 19.0, IQR: 17.0-21.0) than those without ASD (median 11.0, IQR: 9.0-15.0), with a median difference of 8.0 points (95% CI: 6.0-10.0).

**Conclusion:**

A significant proportion of mothers of preterm infants screened positive for acute stress disorder and postpartum depression hence, Preterm delivery appears to be a traumatic event, increasing the risk of stress-related and depressive disorders in mothers.

## INTRODUCTION

A preterm birth occurs before 37 weeks of gestation.^1^ Advances in neonatal intensive care have resulted in dramatically higher survival rates for preterm neonates. Preterm birth is still associated with considerable childhood mortality and morbidity.^2^ Having a newborn child admitted to the Neonatal Intensive Care Units (NICU) is an unexpected and frightening experience for parents.^3^ Parents may be concerned about their child’s health and survival, in addition to experiencing separation from their newborn, expected issues of accessing information and talking with the medical staff.^4^ According to research, bonding with newborns begins before delivery and evolves afterward however, if the birth arrives sooner than planned, the usual bonding process may be disrupted.^5^ The experience of a preterm delivery affects parents’ emotional health both immediately and over time, which may influence the child’s long-term development.^6^

Although the NICU has experienced considerable adjustments in the recent decade to facilitate the presence of parents throughout their baby’s hospitalisation, the NICU continues to be a stressful place for parents, as proved in several studies.^7^ Previous studies have consistently demonstrated that feelings of parental stress, estrangement and heightened anxiety are widespread.^3^-^8^ According to studies, parents who experience stressful events such as NICU/pediatric intensive care unit (PICU) admission and pediatric traumas may exhibit symptoms of Acute stress disorder (ASD). The DSM-5 describes ASD as the development of specific fear behaviors that last from three days to one month after a traumatic event. Diagnostic criteria for ASD involve symptoms of dissociation, reexperiencing, avoidance, and hyperarousal that cause significant impairment to everyday functioning. ASD has been used as a marker to reasonably predict post-traumatic stress disorder (PTSD).^9^ Postpartum ASD was diagnosed in 14.9% of mothers and 4.8% in fathers with very low birth weight infants, but in no mother or father with term infants.^10^ In another study on the prevalence of posttraumatic stress in parents of infants in the NICU, it was found that 35% of mothers and 24% of fathers met the ASD diagnostic criteria.^11^

Our research aimed to investigate the frequency of ASD and postnatal depression in mothers with preterm infants admitted to the NICU. The significant proportion, with approximately 30-40 % of monthly preterm admissions in our NICU, underscores the relevance and urgency of understanding the psychological consequences for mothers in this specific population.

## METHODOLOGY

The is a cross-sectional study done in a 24-bed level-3 NICU at Aga Khan University Hospital in Karachi. The study was conducted between July 2024 to February 2025.

### Ethics Statement:

Informed written consent was obtained from each participant prior to data collection. All data were collected anonymously and kept confidential in accordance with the Declaration of Helsinki guidelines.

### Ethical Approval:

This study was reviewed and approved by the Ethics Review Committee of Aga Khan University, Karachi, Pakistan (ERC Reference No.: 2024-9927-29911).

### Inclusion Criteria:

Mothers of preterm newborns below 34 weeks’ gestational age admitted to the NICU, including multiple births, who offer written informed consent.

### Exclusion Criteria:

Mothers whose infants had known fetal abnormalities or a serious disease that required compassionate care, as evaluated by the Physician NICU. Mothers who were not visiting their infants due to any medical condition or had been diagnosed with a mental health illness and at that time taking antidepressant, as these characteristics might alter maternal-infant contact and affect study outcomes.

### Sample Size:

The sample size was calculated using OpenEpi, version 3. A comparable survey found that 34.9% of preterm mothers were diagnosed with ASD.^11^ The sample size was determined to be 119. However, research on postnatal depression found that the prevalence of postnatal depression ranges between 3.5% and 40%. 12 Hence, the ultimate sample size is 123.

### Sampling Method:

Study participants were conveniently sampled through a Structured Interview.

### Data Collection:

Interviews were caried out 2-4 weeks after admission to NICU. The research team used validated Stanford Acute Stress Reaction Questionnaire (SASRQ) for (ASD) and the Edinburgh Postnatal Depression Scale (EPDS) for Depression. Participants filled up a demographic questionnaire. Data on newborn characteristics such as gestational age, birth weight, Apgar scores, and length of stay in the NICU were retrieved from medical records.

### Statistical Analysis:

The data were analysed using PSPP software. Descriptive statistics were utilised to sum up maternal and neonatal parameters. Continuous variables were measured using means and standard deviations, whilst categorical data were reported using frequencies and percentages. Associations between potential predictors and outcomes (ASD and postpartum depression) were evaluated using chi-square tests.

## RESULTS

For this study, 123 premature babies were enrolled.

### Participants:


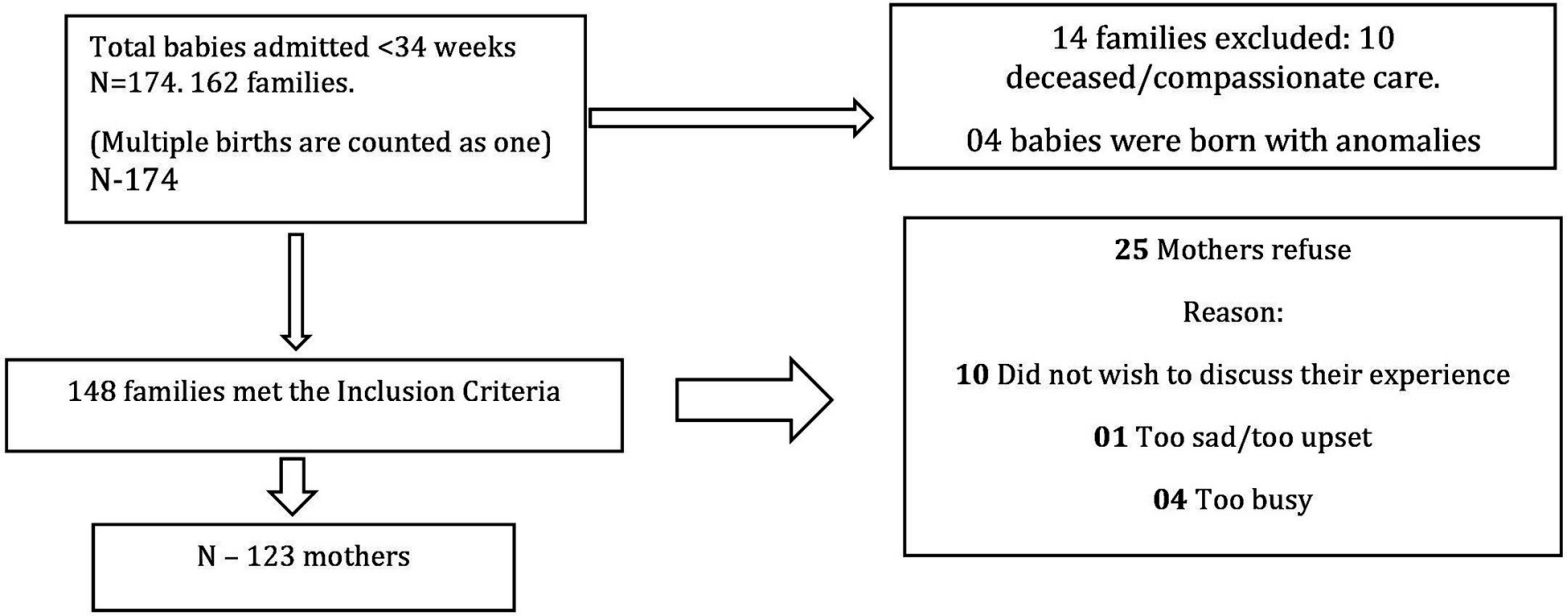


### Descriptive Data:

For this study, 123 premature babies were enlisted. The mean and SD were used to summarize continuous variables for descriptive analysis, whereas frequencies and percentages were used to report the categorical variables.

### Outcome data:

### Baseline characteristics of Preterm Neonates:

The baseline attributes of preterm neonates as shown in Table-I. With an IQR of 29.6 to 33.0 weeks and a median gestational age of 32.0 weeks. The majority of them (57.7%) were moderately preterm (32 - <37 weeks). Apgar scores are a rapid indicator of a newborn’s physical health just after birth. Median values and IQRs are reported for 1 and 10 minutes post-delivery. At one minute after delivery, the median Apgar score was 7.0 (IQR: 6.0-8.0), and ten minutes later, it improved to 9.0 (IQR: 8.0-9.0). The average birth weight was 1.6 ± 0.5 kg, which is in line with (Table-I) overall preterm status. Additionally, it revealed that the period of supportive care needed varied, with a median of 14.0 days and an IQR of 7.0 to 20.0 days for NICU hospitalization. While 51.2% of infants needed extended hospital admissions, almost 60 (48.8%) had NICU stays that were comparatively shorter, suggesting a quicker clinical recovery or less need for intensive assistance.

**Table-I:** Baseline characteristics of Infants.

Variables	Range
Baseline Characteristics of Infants N=123	n %
Gestational Age in Weeks, Median (IQR)	32.0 (29.6-33.0)
Extremely preterm (<28 weeks)	18 (14.6%)
Very preterm (28 - <32 weeks)	34 (27.6%)
Moderate preterm (32 - <37 weeks)	71 (57.7%)
Apgar Score at (1 min), Median (IQR)	7.0 (6.0-8.0)
Apgar Score at (10 min), Median (IQR)	9.0 (8.0-9.0)
Baby’s Weight at birth in KG, Mean ± SD	1.6 ± 0.5
Days in NICU, Median (IQR)	14.0 (7.0-20.0)
< 13 days	60 (48.8%)
≥ 13 days	63 (51.2%)

### Gynaecological and Sociodemographic Characteristics of the mothers of Preterm babies:

The mean age of the mothers was 30.6 ± 5.3 years. The median duration of marriage was 4.0 years (IQR: 2.0-6.0), with 69.1% being married for three or more years. Among 123 respondents, the mean number of children was 1.0 ± 1.2, with a median of 1.0 (IQR: 0.0-2.0). The mean number of living children was 0.8 ± 1.1, and of deceased children was 0.1 ± 0.5. A history of child death was reported by 8.1% of participants, while 32.5% reported a history of miscarriage. Premature birth had occurred in 5.7%, and NICU admission was reported by 4.1%. A history of trauma was present in 15.4% of the mothers. The majority of mothers were graduates (47.2%). Regarding household income, 75.6% reported a monthly income of more than PKR 200,000.

### Psychological Health Status of Mothers:

The median EPDS score was 15.0 (IQR: 10.0-19.0). Based on EPDS scores, 54.5% of mothers screened positive for postnatal depression, while 45.5% did not. The median SASRQ score was 36.0 (IQR: 19.0-70.0). ASD was present in 43.1% of mothers. Around 36.6% of mothers had both ASD and postnatal depression, as displayed in (Table-II).

**Table-II:** Sociodemographic Variables of Mothers and Mental Health Outcomes of the Study Participant.

Mother’s Age (yrs), Mean ± SD	30.6 ± 5.3
18-29 years old	60 (48.8%)
30-59 years old	63 (51.2%)
Married Since, Median (IQR)	4.0 (2.0-6.0)
< 3 years	38 (30.9%)
≥ 3 years	85 (69.1%)
No. of Children, Mean ± SD (n=122)	1.0 ± 1.2
No. of Children, Median (IQR) (n=122)	1.0 (0.0-2.0)
Alive, Mean ± SD	0.8 ± 1.1
Alive, Median (IQR)	0.0 (0.0-1.0)
Deceased, Mean ± SD	0.1 ± 0.5
Deceased, Median (IQR)	0.0 (0.0-0.0)
***Child Death History***	
No	113 (91.9%)
Yes	10 (8.1%)
***Miscarriage History***	
No	83 (67.5%)
Yes	40 (32.5%)
***Premature Birth History***	
No	116 (94.3%)
Yes	7 (5.7%)
***NICU Admission History***	
No	118 (95.9%)
Yes	5 (4.1%)
***Trauma History***	
No	104 (84.6%)
Yes	19 (15.4%)
***Education Level***	
Matric	3 (2.4%)
Intermediate	12 (9.8%)
Undergraduate	21 (17.1%)
Graduate	58 (47.2%)
Masters	29 (23.6%)
***Employed***	
No	91 (74.0%)
Yes	32 (26.0%)
***Family Income (PKR)***	
< 50,000	2 (1.6%)
50,000-100,000	5 (4.1%)
100,000-200,000	23 (18.7%)
> 200,000	93 (75.6%)
Edinburgh Postnatal depression Scale (EPDS) Total Score, Median (IQR)	15.0 (10.0-19.0)
No	56 (45.5%)
Yes	67 (54.5%)
***Most Disturbing Event Level***	
Not at all disturbing	6 (4.9%)
Somewhat disturbing	31 (25.2%)
Moderately disturbing	15 (12.2%)
Very disturbing	39 (31.7%)
Extremely disturbing	32 (26.0%)
Stanford Acute Stress Reaction Questionnaire (SASRQ) Total Score, Median (IQR)	36.0 (19.0-70.0)
***Acute Stress Disorder (ASD)***	
No	70 (56.9%)
Yes	53 (43.1%)
***Postnatal depression***	
ASD With EPDS	45 (36.6%)
ASD Without EPDS	8 (6.5%)
EPDS Without ASD	22 (17.9%)
No ASD & EPDS	48 (39.0%)
No. of days pt experienced symptoms of distress, Median (IQR)	5.0 (3.0-5.0)
***Gender***	
Boy	82 (66.7%)
Girl	41 (33.3%)

**Table-III:** Factors associated with Acute Stress Disorder.

Factors associated with Acute Stress Disorder (ASD), N=123	Yes	No	p-value
	53 (43.1%)	70 (56.9%)	
Mother’s Age (yrs), Mean ± SD	30.8 ± 5.8	30.5 ± 4.9	0.79
18-29 years old	29 (54.7%)	31 (44.3%)	0.25
30-59 years old	24 (45.3%)	39 (55.7%)	
Married Since, Median (IQR)	5.0 (3.0-7.0)	3.0 (2.0-5.0)	0.19
< 3 years	13 (24.5%)	25 (35.7%)	0.18
≥ 3 years	40 (75.5%)	45 (64.3%)	
No. of Children, Mean ± SD, n= 122	1.0 ± 1.2	0.9 ± 1.1	0.72
No. of Children, Median (IQR), n= 122	1.0 (0.0-2.0)	1.0 (0.0-2.0)	0.82
Alive, Mean ± SD	0.9 ± 1.1	0.8 ± 1.1	0.49
Alive, Median (IQR)	1.0 (0.0-2.0)	0.0 (0.0-1.0)	0.42
Deceased, Mean ± SD	0.2 ± 0.5	0.1 ± 0.4	0.37
Deceased, Median (IQR)	0.0 (0.0-0.0)	0.0 (0.0-0.0)	0.4
Child Death History			0.33
No	47 (88.7%)	66 (94.3%)	
Yes	6 (11.3%)	4 (5.7%)	
Miscarriage History			0.025
No	30 (56.6%)	53 (75.7%)	
Yes	23 (43.4%)	17 (24.3%)	
Premature Birth History			0.14
No	48 (90.6%)	68 (97.1%)	
Yes	5 (9.4%)	2 (2.9%)	
NICU Admission History			0.65
No	50 (94.3%)	68 (97.1%)	
Yes	3 (5.7%)	2 (2.9%)	
Trauma History			0.015
No	40 (75.5%)	64 (91.4%)	
Yes	13 (24.5%)	6 (8.6%)	
Education Level			0.93
Matric	1 (1.9%)	2 (2.9%)	
Intermediate	5 (9.4%)	7 (10.0%)	
Undergraduate	10 (18.9%)	11 (15.7%)	
Graduate	23 (43.4%)	35 (50.0%)	
Masters	14 (26.4%)	15 (21.4%)	
Employed			<0.001
No	30 (56.6%)	61 (87.1%)	
Yes	23 (43.4%)	9 (12.9%)	
Family Income			0.41
< 50,000	2 (3.8%)	0 (0.0%)	
50,000-100,000	3 (5.7%)	2 (2.9%)	
100,000-200,000	9 (17.0%)	14 (20.0%)	
> 200,000	39 (73.6%)	54 (77.1%)	
Gestational Age in Weeks, Median (IQR)	29.9 (27.6-31.0)	33.0 (32.0-33.0)	<0.001
Preterm			<0.001
Extremely preterm (<28 weeks)	14 (26.4%)	4 (5.7%)	
Very preterm (28 - <32 wks)	27 (50.9%)	7 (10.0%)	
Moderate preterm (32 - <37 wks)	12 (22.6%)	59 (84.3%)	
Baby’s Weight in KG, Mean ± SD	1.3 ± 0.4	1.8 ± 0.5	<0.001
Days in NICU, Median (IQR)	20.0 (12.0-28.0)	10.0 (7.0-15.0)	<0.001
< 13 days	14 (26.4%)	46 (65.7%)	<0.001
≥ 13 days	39 (73.6%)	24 (34.3%)	
Apgar Score at (1 min), Median (IQR)	6.0 (5.0-8.0)	7.0 (7.0-8.0)	0.021
Apgar Score at (10 min), Median (IQR)	8.0 (7.0-9.0)	9.0 (9.0-9.0)	<0.001
Edinburgh Postnatal depression Scale (EPDS) Total Score, Median (IQR)	19.0 (17.0-21.0)	11.0 (9.0-15.0)	<0.001
No	8 (15.1%)	48 (68.6%)	<0.001
Yes	45 (84.9%)	22 (31.4%)	
Most Disturbing Event Level (1-5)			<0.001
Not at all disturbing	0 (0.0%)	6 (8.6%)	
Somewhat disturbing	5 (9.4%)	26 (37.1%)	
Moderately disturbing	4 (7.5%)	11 (15.7%)	
Very disturbing	18 (34.0%)	21 (30.0%)	
Extremely disturbing	26 (49.1%)	6 (8.6%)	
Postnatal depression			<0.001
ASD With EPDS	45 (84.9%)	0 (0.0%)	
ASD Without EPDS	8 (15.1%)	0 (0.0%)	
EPDS Without ASD	0 (0.0%)	22 (31.4%)	
No ASD & EPDS	0 (0.0%)	48 (68.6%)	
No. of days pt experienced symptoms of distress, Median (IQR)	6.0 (5.0-6.0)	3.0 (2.0-4.0)	<0.001
Gender			0.52
Boy	37 (69.8%)	45 (64.3%)	
Girl	16 (30.2%)	25 (35.7%)	

A significant association (<0.025) between miscarriage history and ASD. Mothers who had experienced miscarriage were more likely to develop ASD, possibly due to the cumulative psychological burden. ASD was substantially more common in mothers of preterm infants, particularly those born extremely or very preterm. Mothers with ASD were also more likely to suffer from postnatal depression (<0.001). Most mothers with ASD (84.9%) also obtained high scores on the EPDS. This highlights the overlap between ASD and depressive symptoms postpartum. Longer NICU stays, especially stays ≥13 days, significantly increase ASD risk (<0.001), possibly due to prolonged maternal stress, uncertainty, and separation from the infant. At one and ten minutes, the ASD group’s median Apgar scores were lower, indicating worse neonatal outcomes.

**Fig.1:**
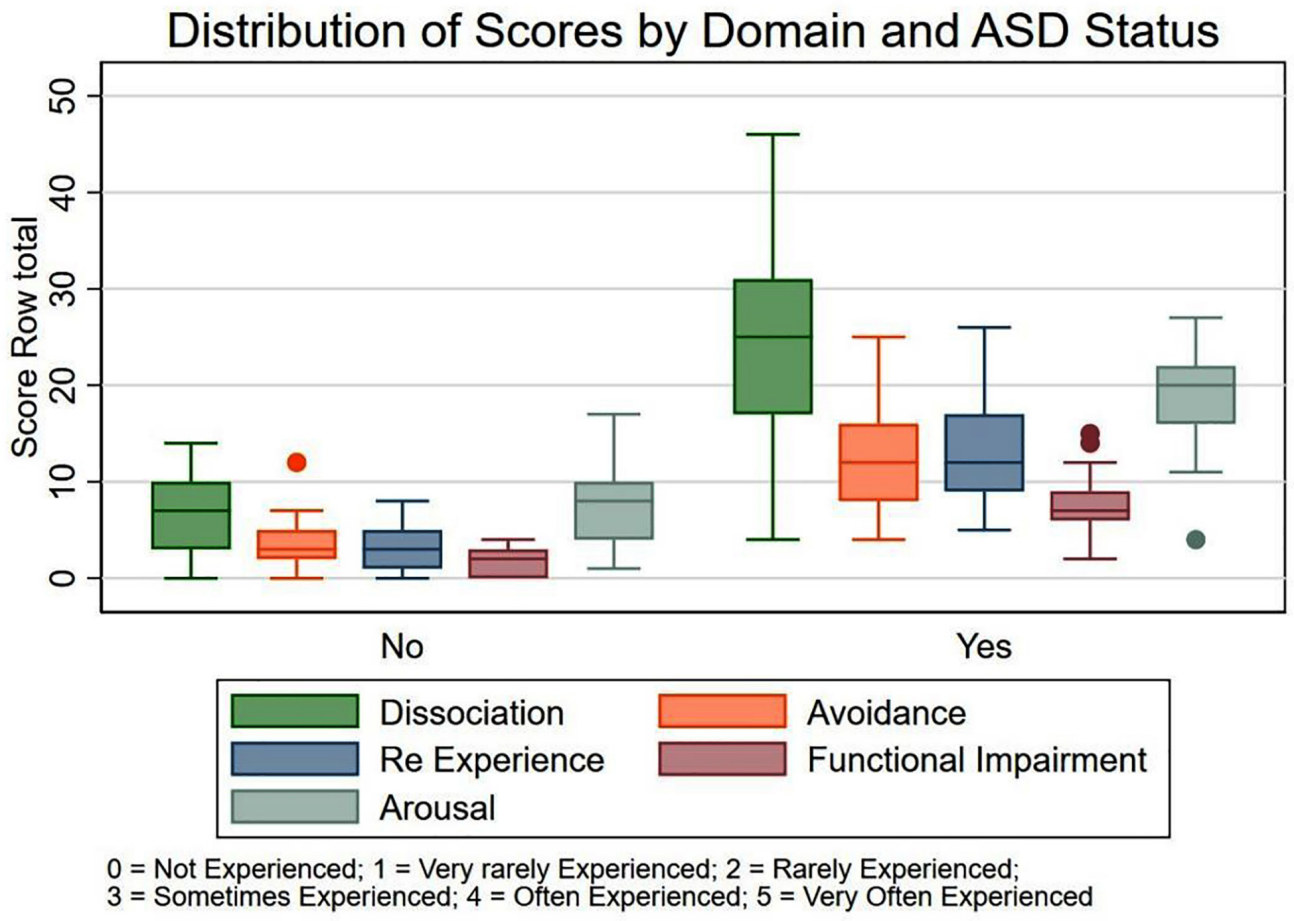
Frequency of ASD symptoms among mothers.

A statistically significant (<0.001) relationship was found that prior exposure to trauma may predispose mothers to acute stress reactions following childbirth-related stressors. Nearly half of the mothers with ASD rated the most upsetting incident as “extremely disturbing,” indicating higher levels of distress. ASD was substantially correlated with employment, which may be a reflection of added stress from juggling job and caring obligations. Participants with ASD report much greater and more varied symptom severity across all categories, particularly dissociation and re-experience (Fig.1).

**Fig.2:**
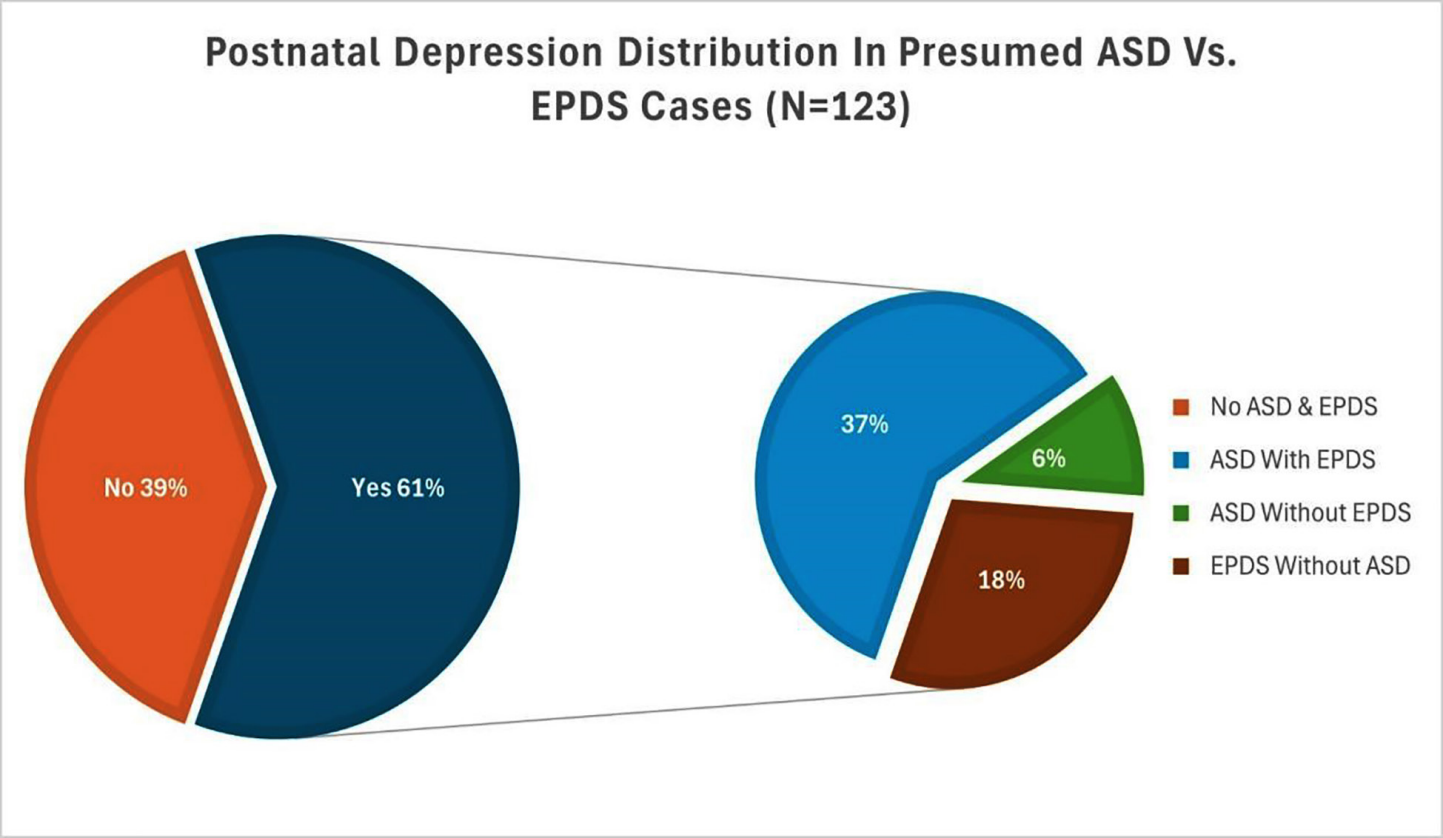
Postnatal Depression Distribution in Presumed ASD vs. EPDS Cases.

The majority of postpartum mothers (61%) reported having clinically severe depressive symptoms, as seen in (Fig.2). A significant psychological load was indicated by the fact that more than one-third (37%) had both postnatal depression and ASD. These results emphasize the necessity of early intervention and frequent screening in the postnatal period.

## DISCUSSION

This study fills a major gap in the local literature to evaluate the prevalence of postnatal depression and ASD in mothers of preterm newborns admitted to the NICU in the Pakistani population. There is a significant paucity of Pakistani research on the NICU experience as a parental stressor. The average maternal age was 30.6 years, with about equal distribution between the younger (18-29 years; 48.8%) and older (30-59 years; 51.2%) age groups. The median marital duration of four years, with 69.1% of women married for ≥3 years, suggests a solid marital context, which is frequently considered protective against prenatal mental health issues. Despite this, a large proportion of mothers (54.5%) tested positive for postnatal depression using the EPDS and 43.1% fulfilled criteria for ASD using the SASRQ.

The dissociation and arousal domains had significantly higher median and interquartile range values, indicating frequent and varied stress-related symptoms in these regions. Prior obstetric hardship was not rare, despite the comparatively low average number of children per mother (Mean = 1.0 ± 1.2) and the infrequent reporting of child loss (8.1%). Approximately one-third (32.5%) had experienced a miscarriage, while 5.7% and 4.1% reported premature births and NICU admissions, respectively. Previous study proved that the incidence of ASD/PTSD has been associated with psychosocial stressors, trauma exposure, and demographic characteristics.^12^ Although delivering a baby is typically a happy event, many postpartum women develop depressive symptoms and disorders.^13^ According to the American Psychiatric Association’s Diagnostic and Statistical Manual (DSM-5), onset of postpartum major depression can occur prior to or after parturition.^14^

The DSM-5 specifier “with peripartum onset” is used when the onset of major depression occurs either during pregnancy or in the four weeks following delivery. According to randomized control trials reasonable time to screen is four to eight weeks after delivery.^15^-^16^ Holditch and associates found significant depressive symptoms in 40% of mothers of very premature infants compared with 10% to 15% of the population.^17^ According to another study, the prevalence of postnatal depression varies significantly. Due to time restrictions and concerns about the social acceptability of screening, these illnesses, despite their frequency, are typically neither recognized nor treated.^18^ In this cross-sectional study we used the SASRQ and EPDS which are well validated instruments to ensure accurate measurement of acute stress and postpartum depression.

The SASRQ identifies acute stress symptoms across dissociation, re-experiencing, avoidance, arousal, and functional impairment. The requirement of multiple dissociative symptoms (≥3) increases diagnostic specificity and authenticity.^19^-^20^ EPDS has been considered extensively validated for postpartum populations of Asia.^21^ According to Stefanie Zaers et al.’s study, 22% of women experienced depression symptoms six weeks after giving birth.^22^ According to a study conducted in Iran by Naeem, Azam Naeem et al., 32.5% of mothers experienced ASD shortly after birth, with 40% developing PTSD a month later.^23^ Allison Baylor Williams et al. discovered that about 55 percent of NICU mothers had ASD symptoms.^24^

Our study suggests that socio-demographic indicators further contextualise the findings, which goes against the usual trends that emphasise the universality of postpartum emotional distress and challenge the conventional wisdom that perinatal mental health burdens are more common in socioeconomically disadvantaged populations. The majority of mothers (70.8%) were educated at the graduate level or above, and over three-quarters (75.6%) came from households with higher incomes (>PKR 200,000), indicating that psychological morbidity was not limited to low-resource groups’ distress. According to our study’s results, 61% of participants had positive results for postnatal depression on the EPDS. According to research by Anna Alkozei et al., 38% of mothers exhibited major depression symptoms (EPDS score ≥10) and 52% of mothers reported higher levels of stress (PSS: NICU score ≥3). The biggest cause of stress among NICU mothers was stress associated with changes in their parental roles.^25^

In a Ghanaian cohort, John Pellegrino et al. employed the Perceived Stress Scale-4 (PSS-4) and the Patient Health Questionnaire-9 (PHQ-9) to determine that 43.5% of the population had postpartum stress, whereas 3.9% of the population had moderate to moderately severe postpartum depression.^26^ According to a study by Kameelah Gateau et al., which involved a majority of Hispanic, non-English speaking individuals, 33% of mothers tested positive for acute PTS.^27^ According to a study by Jubinville et al., 43% of mothers of premature infants in the NICU experienced severe depressive symptoms 7-10 days after the baby was born, and the symptoms persisted for a month.^28^

### Strengths:

This study ensures data dependability by using standardised screening instruments in a tertiary care context. Additionally, focusing on an under-researched population highlights important cultural and contextual factors relevant to maternal mental health in low- and middle-income countries.

### Limitations:

A cross-sectional study design was employed. However, longitudinal or prospective cohort studies may yield better results. The study has limited generalisability.

## CONCLUSION

Recognizing ASD in the early stages is crucial, as it can potentially impact the long-term mental health outcomes for both mothers and their infants. The premature birth experience is traumatic for mothers and may lead to various emotional responses, including stress-related symptoms such as postnatal depression and/or ASD. Mothers with significant symptoms of depression and those with symptoms of stress are more at risk for developing symptoms of ASD followed by PTSD. These findings encourage the inclusion of mental health services in programs for the care of mothers and highlight the importance of routine psychological screening throughout the postnatal period, irrespective of socioeconomic position. Importantly, the presence of ASD alone, or in combination with depression, may impair maternal-infant bonding and breastfeeding, and affect long-term neurodevelopmental outcomes for the infant. Therefore, early recognition and intervention are essential.

## Authors’ Contribution:

**SF:** Conceived, designed and did statistical analysis & editing of manuscript, is responsible for integrity of research.

**SF, MC and VNAB:** Did data collection and manuscript writing.

**AS:** Did critical review and final approval of manuscript..

## References

[1] Quinn JA, Munoz FM, Gonik B, Frau L, Cutland C, Mallett-Moore T (2016). Preterm birth: Case definition &guidelines for data collection, analysis, and presentation of immunization safety data. Vaccine.

[2] Garg D, Chaudhury S, Saldanha D, Kumar S (2023). Stress, postpartum depression, and anxiety in mothers of neonates admitted in the NICU: A cross-sectional hospital-based study. Ind Psychiatry J.

[3] Shaw RJ, Deblois T, Ikuta L, Ginzburg K, Fleisher B, Koopman C (2006). Acute stress disorder among parents of infants in the neonatal intensive care nursery. Psychosomatics.

[4] Malouf R, Harrison S, Burton HA, Gale C, Stein A, Franck LS (2022). Prevalence of anxiety and post-traumatic stress among parents of babies admitted to neonatal units: A systematic review and meta-analysis. EClinicalMedicine.

[5] Goldberg S, DiVitto B Parenting children born preterm. (Suspicious) recheck this reference.

[6] Howard K, Martin A, Berlin LJ, Brooks-Gunn J (2011). Early mother-child separation, parenting, and child well-being in Early Head Start families. Attach Hum Dev.

[7] Franck LS, Cox S, Allen A, Winter I (2005). Measuring NICU-related parental stress. J Adv Nurs.

[8] Vanderbilt D, Bushley T, Young R, Frank DA (2009). Acute posttraumatic stress symptoms among urban mothers with newborns in the NICU: A preliminary study. J Dev Behav Pediatr.

[9] Substance Abuse and Mental Health Services Administration (2016). Impact of the DSM-IV to DSM-5 Changes on the National Survey on Drug Use and Health.

[10] Helle N, Barkmann C, Ehrhardt S, Bindt C (2018). Postpartum posttraumatic and acute stress in mothers and fathers of infants with very low birth weight. J Affect Disord.

[11] Lefkowitz DS, Baxt C, Evans JR (2010). Prevalence and correlates of posttraumatic stress and postpartum depression in NICU parents. J Clin Psychol Med Settings.

[12] Kalar MU, Fatima I, Nabila K, Zainab A, Wardah G, Zara R (2012). Prevalence and predictors of postnatal depression in mothers of Karachi. Int J Collab Res Intern Med Public Health.

[13] Howard LM, Molyneaux E, Dennis CL, Rochat T, Stein A, Milgrom J (2014). Non-psychotic mental disorders in the perinatal period. Lancet.

[14] American Psychiatric Association (1994). Diagnostic and Statistical Manual of Mental Disorders: DSM-IV.

[15] O'Connor E, Rossom RC, Henninger M, Groom HC, Burda BU (2016). Primary care screening for and treatment of depression in pregnant and postpartum women: Systematic review. JAMA.

[16] Reynolds CF, Frank E (2016). USPSTF recommendation statement on screening for depression in adults: Not good enough. JAMA Psychiatry.

[17] Holditch-Davis D, Bartlett TR, Blickman AL, Miles MS (2003). Posttraumatic stress symptoms in mothers of premature infants. J Obstet Gynecol Neonatal Nurs.

[18] Bronner MB, Kayser AM, Knoester H, Bos AP, Last BF, Grootenhuis MA (2009). Peritraumatic dissociation and coping styles as risk factors for posttraumatic stress, anxiety, and depression in parents after PICU admission. Child Adolesc Psychiatry Ment Health.

[19] Bano A, Shahdi R, Riaz R, Ali S, Husain S (2023). Mental health, stress reaction and suicidal ideation among adolescent offspring of alcoholic fathers. OEconomia.

[20] Lötvall R, Palmborg Å, Cardeña E (2022). A 20-year review of the Stanford Acute Stress Reaction Questionnaire. Eur J Trauma Dissociation.

[21] Cox JL, Holden JM, Sagovsky R (1987). Detection of postnatal depression: Development of the Edinburgh Postnatal Depression Scale. Br J Psychiatry.

[22] Zaers S, Waschke M, Ehlert U (2008). Depressive symptoms and PTSD symptoms after childbirth. J Psychosom Obstet Gynaecol.

[23] Naeem AT, Nayeri F, Shariat M, Zarkesh MR, Abedinia N, Bakhsh ST (2019). Incidence and risk factors of PTSD among parents of NICU-hospitalized preterm neonates. Iran J Neonatol.

[24] Williams AB, Hendricks-Muñoz KD, Parlier-Ahmad AB, Griffin S, Wallace R, Perrin PB (2021). Posttraumatic stress in NICU mothers. J Perinatol.

[25] Alkozei A, McMahon E, Lahav A (2014). Stress levels and depressive symptoms in NICU mothers in early postpartum. J Matern Fetal Neonatal Med.

[26] Pellegrino J, Mundagowa PT, Sakyi KS, Owusu PG, Agbinko-Djobalar B, Larson LM (2025). Prevalence and risk factors for postpartum depression and stress among mothers of preterm infants in Accra. Int J Gynaecol Obstet.

[27] Gateau K, Song A, Vanderbilt DL, Gong C, Friedlich P, Kipke M (2021). Maternal post-traumatic stress and depression after NICU discharge. BMC Pregnancy Childbirth.

[28] Jubinville J, Newburn-Cook C, Hegadoren K, Lacaze-Masmonteil T (2012). Symptoms of acute stress disorder in mothers of premature infants. Adv Neonatal Care.

